# Antipsychotic Treatment Reduces Indices of Oxidative Stress in First-Episode Psychosis Patients

**DOI:** 10.1155/2016/9616593

**Published:** 2016-07-27

**Authors:** Kärt Kriisa, Liina Haring, Eero Vasar, Kati Koido, Sven Janno, Veiko Vasar, Kersti Zilmer, Mihkel Zilmer

**Affiliations:** ^1^Institute of Biomedicine and Translational Medicine, Department of Biochemistry, The Centre of Excellence for Genomics and Translational Medicine, Faculty of Medicine, University of Tartu, 19 Ravila Street, 50411 Tartu, Estonia; ^2^Psychiatry Clinic, Tartu University Hospital, 31 Raja Street, 50417 Tartu, Estonia; ^3^Institute of Biomedicine and Translational Medicine, Department of Physiology, The Centre of Excellence for Genomics and Translational Medicine, Faculty of Medicine, University of Tartu, 19 Ravila Street, 50411 Tartu, Estonia

## Abstract

38 first-episode psychosis (FEP) patients and 37 control subjects were recruited for the study of indices of oxidative stress (OxS). The main purpose of the study was to compare the OxS statuses (serum total antioxidant capacity (TAC), total level of peroxides (TPX), oxidative stress index (OSI), and ratio oxidized methionine (Met-SO) to methionine (Met)) between antipsychotic-naïve FEP patients and individuals without a history of psychiatric disorders. Subsequently, the impact of 7-month antipsychotic treatment was evaluated on the OxS status in FEP patients. An attempt was made to assess links between OxS signature and inflammation markers. The oxidative stress indices remained generally unchanged in antipsychotic-naïve FEP patients compared to control subjects. Despite that, there was a significant correlation between the levels of TPX and EGF (endothelial growth factor) in FEP patients. This correlation disappeared after antipsychotic treatment of FEP patients. Moreover, antipsychotic treatment was associated with a significant reduction in OxS indices, including TPX, OSI, and ratio between Met-SO and Met. By contrast, in chronic SCZ patients we established a significant high-grade OxS. In conclusion, the markers of total antioxidative capacity, lipid peroxidation, and protein oxidation revealed no high-grade OxS in FEP patients. Nevertheless, antipsychotic treatment induced a considerable anti-inflammatory effect. OxS levels were also significantly decreased if compared in FEP patients before and after antipsychotic treatment.

## 1. Introduction

Schizophrenia (SCZ) is a complex, heterogeneous, and severe psychiatric illness that affects about 1% of the population [1]. Exact molecular mechanisms underlying the pathogenesis of this disorder remain to be elucidated. Preclinical and clinical studies of the last decade have highlighted a number of data demonstrating the involvement of OxS in the pathophysiology of psychiatric diseases [2, 3]. Recent studies support the understanding that both susceptibility to OxS and the status (level) of OxS may underlay the pathogenesis of SCZ via different mechanisms [4, 5].

There is a growing interest in the research of the development and course of first-episode psychosis (FEP) [6]. FEP can be seen as an intermediate state which has important implications for further clinical course, treatment, and management. Several studies [7, 8] suggest that OxS-driven injury occurs at the onset of psychosis. Recent evidence suggests that OxS plays a role in the etiopathogenesis of mental diseases usually starting with FEP [9]. Growing evidence shows that FEP is associated with some features of OxS [10, 11]. Considering the potential role of OxS in the course of FEP, the OxS signature of FEP patients should be studied by measuring OxS-related parameters in comparison with carefully selected mentally healthy subjects. To describe the blood overall status (level) of OxS, it is preferable to use markers that are clinically easy to apply.

Several studies show links between inflammation and OxS [12, 13]. Recently we demonstrated [14] that antipsychotic-naïve FEP patients display increased indices of elevated low-grade inflammation. Antipsychotic therapy resulted in significant clinical improvement of psychotic symptoms and decline in inflammatory status. The main purpose of this study was to compare the OxS statuses (serum total antioxidant capacity (TAC), total level of peroxides (TPX), oxidative stress index (OSI), and ratio oxidized methionine (Met-SO) to methionine (Met)) between antipsychotic-naïve FEP patients and individuals without a history of psychiatric disorders. Subsequently, the impact of 7-month antipsychotic treatment was evaluated on the OxS status in FEP patients. An attempt was made to assess the link between OxS and inflammation markers.

## 2. Patients and Methods

### 2.1. Participants

38 FEP patients (21 males, 17 females; mean age 25.4 ± 0.89 years) were recruited from the Psychiatric Clinic, Tartu University Hospital, Estonia. They fulfilled the following inclusion criteria: age between 18 and 45; experience of the first psychotic episode; duration of untreated psychosis less than 3 years; no antipsychotic treatment received before the first contact with medical services for psychosis. Patients were excluded from the study when they had psychotic disorders due to a general medical condition or a substance-induced psychosis. FEP diagnoses were based on clinical interview according to ICD-10 [15] criteria. 36 FEP patients completed the follow-up study. Two patients refused to take antipsychotic medications and they were excluded from the follow-up analysis. Antipsychotic history was collected according to the medical chart review. Patients were treated with various antipsychotic medications as clinically indicated. During the follow-up period, patients were receiving antipsychotic medications of either atypical (*n* = 24), typical (*n* = 1), or mixed manner (*n* = 11) and the mean theoretical chlorpromazine dose equivalent was 396 ± 154 mg/day (range 80–640). 28 patients were treated with only antipsychotics, 5 patients additionally needed mood stabilizers, and 6 patients received antidepressants or hypnotics in addition to antipsychotic drugs. Using the information obtained from the participants, we determined that 10 patients and one control subject had used cannabis in their lifetime.

37 healthy subjects participated in the study as control subjects (CS). The sample of CS was recruited by an advertisement from the same geographical area as FEP patients. Both patients and controls were interviewed by experienced psychiatrists in order to avoid the inclusion of subjects with apparent mental disorders as controls. Exclusion criteria for the control group also included psychotic disorder among close relatives. Participants were enrolled between September 2009 and December 2013. The study was approved by the Ethics Committee of University of Tartu, Estonia, and written informed consent was obtained from all participants. The sample of this study contains the same participants as our previous study by Haring et al. [14].

### 2.2. Procedures

For the FEP patients the following activities were conducted at admission and after the follow-up (mean duration 7.18 ± 0.73 months) period: venous blood sampling after a 12-hour overnight fast, application of the PANSS (a rating instrument to evaluate the presence and severity of positive, negative, and general psychopathology, consisting of 30 items, each scored from 1 (absent) to 7 (severe)) [16] for the assessment and clinical monitoring of the disease course and antipsychotic treatment response, and physical examination including evaluating of blood pressure and body mass index (BMI) (weight (kg)/height (m)^2^) data. Blood samples, blood pressure, BMI, and demographic data from CS were collected cross-sectionally.

### 2.3. Blood Collection and Clinical Laboratory Measurements

Blood samples of the participants were collected between 09:00 and 11:00 a.m. Blood (5 mL) was sampled in anticoagulant-free tubes and kept for 1 hour at 4°C (for platelet activation) before serum was isolated (centrifugation at 2000 × 15 minutes at 4°C). Serum was kept at −70°C before testing and all following procedures were performed similarly to our previous studies [17]. The clinical levels of triglycerides, total cholesterol, low-density lipoproteins (LDL cholesterol), high-density lipoproteins (HDL cholesterol), C-reactive protein (CRP), and glycated hemoglobin (HbA1c) were determined by standard laboratory methods using certified assays in the local clinical laboratory. The results of these measurements were presented in our previous study [14].

### 2.4. Oxidative Stress Markers Assay

TPX concentration and TAC of samples were assayed as described previously [18]. TPX concentration of samples was determined using OXYSTAT Assay Kit Cat. Number BI-5007 (Biomedica Gruppe, Biomedica Medizinprodukte GmbH & Co KG, Wien). The kit detects peroxide concentrations based on reaction of the biological peroxides with peroxidase and a subsequent color-reaction using tetramethylbenzidine (TMB) as substrate. After addition of a stop solution, the colored liquid is measured photometrically at 450 nm, using ELISA plate reader Photometer Sunrise (Tecan Austria GmbH, Salzburg). For the assay, a calibrator is used to calculate the concentration of biological peroxides in the sample. The concentration is stated as H_2_O_2_-equivalents (*μ*mol/L). To measure TAC a new more stable coloured 2,2′-azinobis-(3-ethylbenzothiazoline-6-sulfonic acid radical (ABTS^*∗*+^) was employed. The basic principle of the method is that a colorless molecule, reduced ABTS, is oxidized to a characteristic blue-green ABTS^*∗*+^, using hydrogen peroxide in acidic medium (the acetate buffer 30 mml/L pH 3.6). When the colored ABTS^*∗*+^ is mixed with any substance, which can be oxidized, it is reduced to its original colorless ABTS form again. The ABTS^*∗*+^ is decolorized by antioxidants according to their concentrations and antioxidant capacities. The bleaching rate is inversely related with the TAC of sample. This change in color is measured as a change in absorbance at 660 nm. The reaction rate was calibrated with Trolox, which is used as a traditional standard for TAC measurement assays. The results are expressed in mmol Trolox equivalent/L. Within- and between-batch precision data obtained by TAC method were 2.5% and 2.9%, respectively. Percent ratio of the TPX to the TAC is used as OxS index (OSI), an indicator of the degree of OxS [17]. Thus, OSI was calculated as follows: OSI = [(TPX, *μ*mol/L):((TAC, *μ*mol  Trolox/L) × 100)].

Serum level of oxidized methionine (methionine sulfoxide, Met-SO) and methionine (Met) was determined with the AbsoluteIDQ*™* p180 kit (BIOCRATES Life Sciences AG, Innsbruck, Austria) using the flow injection analysis tandem mass spectrometry (FIA-MS/MS) as well as liquid chromatography ((LC)-MS/MS) technique. All measurements were performed as described in the manufacturer's manual UM-P180. Identification and quantification of the metabolites were achieved using multiple reaction monitoring along with internal standards. Calculation of metabolite concentrations was automatically performed by MetIDQ*™* software (BIOCRATES Life Sciences AG).

### 2.5. Cytokine and Growth Factors Assay

The levels of the pro- and anti-inflammatory cytokines of the interleukin (IL-1*α*, IL-1*β*, IL-2, IL-4, IL-6, IL-8, and IL-10) family, tumour necrosis factor-alpha (TNF-*α*), and growth factors, vascular endothelial growth factor (VEGF) and endothelial growth factor (EGF), were measured according to the manufacturer's protocol and the results of these measurements were presented in detail in our previous study [14].

### 2.6. Statistics

Demographic and clinical variables of the FEP patients and CS were compared using analysis of variance or *t*-test for continuous variables and chi-squared test for categorical variables.

The application of Shapiro-Wilk tests indicated that values of oxidative stress markers were not normally distributed (*p* < 0.05). A Mann-Whitney *U* test was applied to compare the raw data of two independent samples (FEP patients before treatment and CS) and a Wilcoxon signed rank test to compare two dependent samples (FEP patients before and after treatment condition). For establishing the effect of treatment, patients were paired one by one.

Spearman's rank correlation analysis was applied to establish the correlations between OxS and low-grade inflammation and metabolic markers, in FEP patients' group, before and after 7-month antipsychotic treatment.

General linear model (GLM) was used to demonstrate the differences in OxS markers levels between antipsychotic-naïve FEP patient and CS. In order to establish treatment effects to OxS serum levels between the groups (FEP patients after 7 months of treatment with antipsychotics) versus CS as well as between subjects (GLM: repeated measures) GLM was utilised. Categorical (disease, gender, and smoking status) and continuous (age) covariates were used in the GLM to compare OxS levels (dependent variables). To study within-subjects' differences in OxS, difference between pre- and posttreatment condition was used as an independent variable. Because GLM analyses required normally distributed data, biomarkers values were log_10_-transformed to approximate normality.

The statistical analyses were performed using Statistica software (StatSoft Inc., 12th Edition) for Windows. All statistical tests were two-sided, and *p* value < 0.05 was considered to be statistically significant.

## 3. Results

### 3.1. General Description of the Study Groups

There were no statistically significant differences between FEP patients (*n* = 38) and CS (*n* = 37) in terms of age (*t*(73) = 0.49, *p* = 0.62) and gender (*χ* ^2^(1) = 1.08, *p* = 0.30). In addition, the differences in tobacco use (8 patients [21.1%] versus 7 controls [18.9%]) were not statistically significant (*χ* ^2^(1) = 0.05, *p* = 0.82). As expected, there was a statistically significant effect after seven months of treatment (Wilcoxon signed rank test, *Z* = 5.23, *p* < 0.000001) on the total symptom score measured by PANSS (median = 112.5, range 80–155 during the recruitment, and median = 62, range 34–100, at the follow-up period). No differences were observed between the 2 samples regarding BMI (Mann-Whitney *U* test, *Z* = −1.01, *p* = 0.31). After 7 months of treatment with antipsychotics, patients BMI was significantly increased (Wilcoxon signed rank test, *Z* = 4.13, *p* < 0.00004).

### 3.2. Differences in Oxidative Stress Markers Levels among Antipsychotic-Naïve FEP Patients and Control Subjects

**Figure 1:**
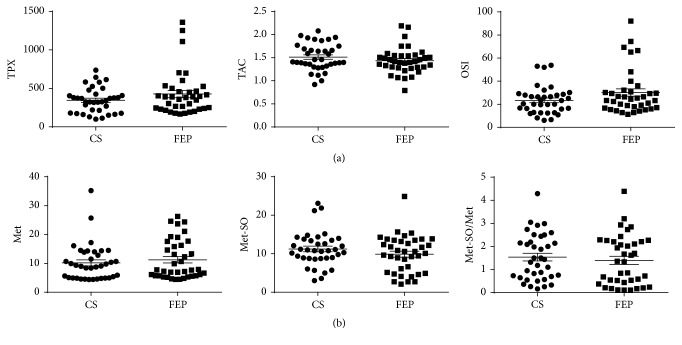
The changes in TPX, TAC, and OSI (a) and Met, Met-SO, and Met-SO/Met (b) due to FEP (Wilcoxon signed rank test). FEP: first-episode psychosis; CS: control subjects.

Regarding OxS-related parameters (TPX, TAC, and OSI) we did not find any difference in FEP patients before treatment compared to CS (Table 1 and Figure 1(a)). Similarly, the levels of Met-SO and Met and the ratio between Met-SO and Met in FEP patients before treatment did not differ from that in CS (Table 1 and Figure 1(b)).

To test the potential effect of the presence of FEP on the combination of OxS markers, we conducted a multivariate GLM analysis. The overall difference between the groups is shown in Table 2. The main effect of the disease emerged on OSI level (*t*_(5,64)_ = 2.26, *p* = 0.03, and *R*_adj_^2^ = 0.17). This effect was accompanied by the main effect of male gender (*t*_(5,64)_ = −3.32, *p* = 0.001). However, interaction between group status and gender regarding OSI level was not statistically significant. At the whole model level, the effect size (partial *η* ^2^) of the disease (before the treatment) on the OxS markers was 0.13 (*F*_(5,64)_ = 1.9, *p* = 0.11).

### 3.3. Antipsychotic Treatment Effect on Biomarkers Levels among FEP Group

**Figure 2:**
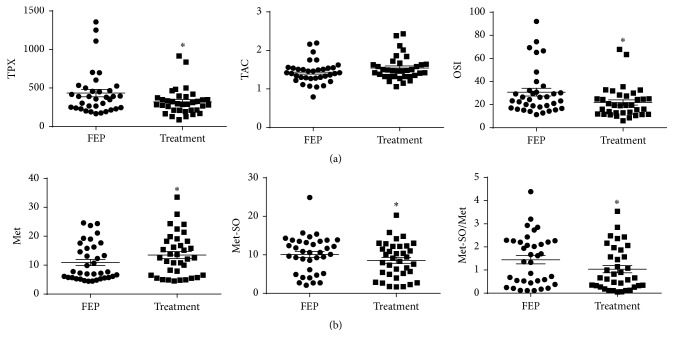
The changes in TPX, TAC, and OSI (a) and Met, Met-SO, and Met-SO/Met (b) due to FEP and antipsychotic treatment. ^*∗*^ *p* < 0.05 (Wilcoxon signed rank test). FEP: first-episode psychosis.

7-month treatment of FEP patients with antipsychotic drugs was associated with a significant decrease in TPX and OSI if compared in FEP patients before therapy and after (Table 1 and Figure 2(a)). Treatment was also associated with a significant increase in Met level and decrease in Met-SO and Met-SO/Met (Table 1 and Figure 2(b)).

On further analyses we evaluated the simultaneous impact of treatment on OxS levels. Repeated measures GLM was performed, to compare the main effects of the 7-month antipsychotic treatment on the serum biomarkers concentrations. The effect of the treatment is summarized in Table 3. TAC, Met, Met-SO, and Met-SO/Met levels were similar between pre- and posttreatment condition while all measured OxS markers were taken into account and a decrease over time was detected in the serum levels of TPX (*p* = 0.04) and OSI (*p* = 0.01). Thus, our results confirm that treatment with antipsychotics has a positive impact on the OxS markers levels during the early phase of the psychotic disease. The effect size (partial *η* ^2^) of the treatment on the OxS markers was 0.17 (*F*_(5,64)_ = 2.00, *p* = 0.08).

Furthermore, to evaluate the FEP patients' posttreatment status regarding OxS markers levels compared to CS (adjusted for age, gender, and smoking status), GLM was performed. As seen from Table 4 levels of TAC, TPX, and OSI in FEP patients group were comparable with CS.

In addition, the 7-month treatment with antipsychotics caused a significant increase in Met (*p* = 0.03) as well as a decrease in Met-SO (*p* = 0.02) and Met-SO/Met (*p* = 0.006) levels in FEP patients' group compared to CS. These changes were associated with effects of age. Treatment effect was significantly associated with higher Met levels in younger patients (*t*_(5,63)_ = −3.06, *p* = 0.003) as well as lower levels of serum Met-SO and Met-SO/Met in older patients (*t*_(5,63)_ = 2.45, *p* = 0.02 and *t*_(5,63)_ = 3.14, *p* = 0.003, resp.).

Spearman rank correlation established a significant positive correlation of TPX with IL-1*β* (*ρ* = 0.26, *p* = 0.03) and EGF (*ρ* = 0.30, *p* < 0.01) in FEP patients' group (Supplementary Table  1 in Supplementary Material available online at http://dx.doi.org/10.1155/2016/9616593). A similar correlation was established for OSI and EGF (*ρ* = 0.24, *p* = 0.04) and for TAC and IL-1*β* (*ρ* = 0.25, *p* = 0.04) (Supplementary Tables  2 and  3). After treatment, we found a significant negative correlation between treatment and OSI (*ρ* = −0.28, *p* = 0.02) and positive correlation between BMI and TAC (*ρ* = 0.31, *p* < 0.01) (Supplementary Tables  4 and  5).

## 4. Discussion

First-episode psychosis can be seen as an intermediate state which has crucial implications for further clinical course, treatment, and management of chronic psychotic disorder. The patients have to receive the best comprehensive science-based management. Therefore, all helpful information (e.g., clinical inspection of actual OxS signature of a patient) for applying contemporary treatment should be taken into account.

Recent articles have shown that OxS plays a role in the etiopathogenesis of psychiatric disorders starting with FEP [9]. Several studies refer to associations between FEP and OxS [10, 11]. However, in order to support these potential associations, further research is needed for verifying such association in FEP patients from different endemic populations and studying the relations between OxS and low-grade inflammation markers. Serum markers of OxS cannot directly reflect OxS status in the brain, but there is data available that changes in serum lipid peroxidation markers are associated with some changes in the brain [19]. TPX, measured in our study, is a well-accepted marker for lipid peroxidation.

Our recent study [14] focused on the characterization of inflammation-related signature of FEP patients before and after 7 months of antipsychotic treatment. Using a carefully selected CS group and the shortest possible time window between the appearance of FEP and collection of blood samples, we tried to capture OxS-related changes resulting from the FEP of schizophrenia. As systemic OxS is a part of the pathogenetic mechanism of chronic psychotic disorder and is causing changes in the entire organism [20], it is reasonable to assess systemic OxS level in the early phase of chronic psychotic disorder.

In this study we did not find significant differences in OxS-related parameters like TPX, TAC, OSI, and Met-SO/Met, when comparing FEP patients with CS (Figures 1(a) and 1(b)). Therefore, total antioxidative capacity, lipid peroxidation, and protein oxidation related indices did not differ in FEP patients before treatment in comparison with CS. Our data are consistent with several studies on FEP [21]. However, one has to note the existence of data showing increased plasma malondialdehyde level in FEP [11]. At the same time the latter study did not show any significant differences in serum TAC. Another recent study also revealed no significant changes in some OxS markers (lipid or protein oxidation or nitric oxide production) in FEP patients compared to CS [10].

Nevertheless, this study underlines that antipsychotic treatment has a double positive impact in FEP patients—simultaneous decrease in OxS-status (Figures 2(a) and 2(b)) with improvement of inflammatory status [14]. However, one has to note that such positive effects of antipsychotic treatment on low-grade inflammation and OxS-status of FEP patients did not continue in long-term chronic schizophrenia patients who developed significant high-grade OxS. In order to support and illustrate the importance of clinical inspection of OxS signature in different stadiums of chronic psychotic disorder, we would give a brief overview of the unpublished results of SCZ patients (*n* = 99) compared to CS (*n* = 51) gained by using the same methodology as in this current study. We found significantly decreased level of TAC (1.43 ± 0.03 and 1.72 ± 0.03, *p* < 0.001 (Mann-Whitney *U* test) in SCZ patients and CS, resp.) and increased levels of TPX (353 ± 21 and 220 ± 9, *p* < 0.0001 (Mann-Whitney *U* test) in chronic SCZ patients and CS, resp.) and OSI (24.4 ± 1.41 and 12.9 ± 0.56, *p* < 0.0001 (Mann-Whitney *U* test) in chronic SCZ patients and CS, resp.) in chronic SCZ patients.

Thus, based on our previous [14] and current data we believe that, already after the diagnosis of FEP, the following treatment strategy has to consider both inflammation and OxS as tightly interrelated counterparts. This statement is supported by the following facts. First, correlations exist between inflammatory and OxS markers in FEP patients [14, Supplementary tables]. Second, antipsychotic treatment, significantly improving the inflammatory signature in FEP patients [14], has a positive impact on the indices of OxS at the early stage of the chronic psychotic disorder. Third, recent reviews and meta-analyses [22–24] focusing on the interactions between inflammation and OxS highlight their tight interrelation in SCZ. Fourth, a recent review emphasized that the administration of EGF to rats causes an elevation of reactive oxygen species and induces behavioural impairments resembling SCZ in rodents. These impairments were significantly improved after applying antioxidative compounds [25]. The established findings are consistent with our previous data concerning the interaction between EGF and inflammation in FEP patients [14] as well as with the effects of antipsychotic treatment on the OxS markers in this study.

The current study had several limitations. First, the limited sample size may create generalizability problems. Although our results should be confirmed in a larger group of patients, they, nevertheless, suggest that there is a need to consider the changes caused by OxS at the early phase of chronic psychotic disorder. Second, we collected data from CS at one time point and did not control their health condition and OxS markers' levels after the same follow-up period, as was done for the FEP patients' group. Third, according to the naturalistic study design, we did not exclude participants who had lifetime exposure to factors such as cannabis misuse or patients who were treated additionally with other psychotropic drugs. Substance misuse [26] as well as the use of antidepressants and mood stabilisers [27, 28] has been associated with effects on OxS status.

## 5. Conclusions

We did not find changes in OxS signature in FEP patients. However, before treatment, TPX levels were significantly correlated with EGF, disappearing after 7 months of antipsychotic medication use. Moreover, the antipsychotic medication of FEP patients not only had a considerable anti-inflammatory effect but also reduced lipid peroxidation and protein oxidation related indices of OxS if compared in FEP patients before and after antipsychotic treatment. By contrast, chronic SCZ displays a signature of high-grade OxS and inflammation.

## Supplementary Material

The following oxidative stress markers and other measured variables were included in Spearman's rank correlation analysis: serum total antioxidant capacity (TAC), total level of peroxides (TPX), oxidative stress index (OSI), methionine (Met), oxidized methionine (Met-SO), ratio oxidized methionine to methionine (Met-SO/Met), pro- and anti-inflammatory cytokines of the interleukin family (IL-1α, IL-1β, IL-2, IL-4, IL-6, IL-8, IL-10), interferon gamma (IFNγ), vascular endothelial growth factor (VEGF), endothelial growth factor (EGF), tumour necrosis factor-alpha (TNF-α), body mass index (BMI), and Positive and Negative Syndrome Scale (PANSS) total score. Supplementary tables 1-2 demonstrate only significant correlation coefficients of the above-mentioned parameters with OxS markers (TAC, TPX, OSI) in antipsychotic-naïve FEP patients' group. Supplementary tables 3-5 present only significant negative or positive correlation coefficients of the above-mentioned parameters with OxS markers (TAC, TPX, OSI) in FEP patients' group after 7-months treatment with antipsychotics.

## Figures and Tables

**Table 1:** Comparisons of oxidative stress markers (OxS) between the first-episode psychosis patients at baseline (FEP_b_) and control subjects (CS) as well as FEP patients at baseline (FEP_b_) and after 7 months of treatment (FEP_f_) with antipsychotics.

OxS markers	CS	FEP_b_	FEP_f_	*Z*-value^a^	*p* value^a^	*Z*-value^b^	*p* value^b^
	Median (min–max)	Median (min–max)	Median (min–max)				
TAC	1.43 (0.92–2.08)	1.42 (0.79–2.19)	1.47 (1.06–2.43)	−1.15	0.25	1.56	0.12
TPX	332.50 (101.00–737.00)	380.00 (166.76–1357.53)	289.00 (88.00–915.55)	1.03	0.30	2.20	0.03
OSI	21.87 (6.16–53.80)	24.38 (11.36–92.02)	19.34 (6.03–67.82)	1.32	0.19	2.56	0.01
Met	9.08 (4.43–35.20)	7.75 (4.46–26.30)	12.50 (4.53–33.50)	0.80	0.43	2.50	0.01
Met-SO	10.80 (3.04–23.10)	10.35 (2.11–24.90)	8.72 (1.69–20.30)	−0.98	0.33	2.05	0.04
Met-SO/Met	1.44 (0.16–4.29)	1.35 (0.11–4.39)	0.66 (0.05–3.54)	−0.86	0.39	2.15	0.03

*Z*-adjusted values^a^ according to Mann-Whitney *U* test (FEP_b_ compared to CS).

*Z*-values^b^ according to Wilcoxon matched pairs test (FEP_b_ compared to FEP_f_).

TAC: total antioxidant capacity; TPX: total level of peroxides; OSI: oxidative stress index; Met: methionine; Met-SO: methionine sulfoxide; Met-SO/Met: ratio of oxidized methionine to methionine.

**Table 2:** Regression coefficients (*β*) and significance values of log_10_-transformed oxidative stress (OxS) markers levels with disease.

Biomarkers	*β*	*β* (95% CI)	*t-*value	*p *value
*OxS markers*				
TAC	−0.13	−0.37–0.11	−1.10	0.28
TPX	0.21	−0.02–0.43	1.82	0.07
OSI	0.25	0.03–0.47	2.26	0.03
Met	0.08	−0.15–0.31	0.70	0.49
Met-SO	−0.18	−0.41–0.05	−1.58	0.12
Met-SO/Met	−0.15	−0.37–0.08	−1.28	0.21

CI: confidence intervals; TAC: total antioxidant capacity; TPX: total level of peroxides; OSI: oxidative stress index; Met: methionine; Met-SO: methionine sulfoxide; Met-SO/Met: ratio of oxidized methionine to methionine.

**Table 3:** Regression coefficients (*β*) and significance values of log_10_-transformed oxidative stress (OxS) markers' levels in first-episode patients' group before treatment compared to log_10_-transformed markers' values measured after 7 months of treatment with antipsychotics.

Biomarkers	*β*	*β* (95% CI)	*t-*value	*p* value
*OxS markers*				
TAC	−0.19	−0.42–0.05	−1.56	0.12
TPX	0.25	0.02–0.48	2.12	0.04
OSI	0.30	0.07–0.53	2.61	0.01
Met	−0.19	−0.43–0.05	−1.62	0.11
Met-SO	0.15	−0.10–0.38	1.21	0.23
Met-SO/Met	0.18	−0.06–0.42	1.51	0.14

CI: confidence intervals; TAC: total antioxidant capacity; TPX: total level of peroxides; OSI: oxidative stress index; Met: methionine; Met-SO: methionine sulfoxide; Met-SO/Met: ratio of oxidized methionine to methionine.

**Table 4:** Regression coefficients (*β*) and significance values of log_10_-transformed oxidative stress (OxS) markers' levels in first-episode patients' group after 7 months of treatment with antipsychotics compared to control subjects.

Biomarkers	*β*	*β* (95% CI)	*t-*value	*p* value
*OxS markers*				
TAC	0.02	−0.22–0.26	0.17	0.87
TPX	−0.01	−0.24–0.21	−0.12	0.90
OSI	−0.02	−0.24–0.20	−0.19	0.85
Met	0.24	0.02–0.47	2.21	0.03
Met-SO	−0.32	−0.54–(−0.09)	−2.77	0.007
Met-SO/Met	−0.31	−0.53–(−0.10)	−2.86	0.006

CI: confidence intervals; TAC: total antioxidant capacity; TPX: total level of peroxides; OSI: oxidative stress index; Met: methionine; Met-SO: methionine sulfoxide; Met-SO/Met: ratio of oxidized methionine to methionine.

## References

[1] http://www.nimh.nih.gov/health/statistics/prevalence/schizophrenia.shtml.

[2] Prabakaran S., Swatton J. E., Ryan M. M. (2004). Mitochondrial dysfunction in schizophrenia: evidence for compromised brain metabolism and oxidative stress. *Molecular Psychiatry*.

[3] Smaga I., Niedzielska E., Gawlik M. (2015). Oxidative stress as an etiological factor and a potential treatment target of psychiatric disorders. Part 2. Depression, anxiety, schizophrenia and autism. *Pharmacological Reports*.

[4] Emiliani F. E., Sedlak T. W., Sawa A. (2014). Oxidative stress and schizophrenia: recent breakthroughs from an old story. *Current Opinion in Psychiatry*.

[5] Tunçel Ö. K., Sarısoy G., Bilgici B. (2015). Oxidative stress in bipolar and schizophrenia patients. *Psychiatry Research*.

[6] Malla A. K., Norman R. M. G., Manchanda R., Townsend L. (2002). Symptoms, cognition, treatment adherence and functional outcome in first-episode psychosis. *Psychological Medicine*.

[7] Mahadik S. P., Mukherjee S., Scheffer R., Correnti E. E., Mahadik J. S. (1998). Elevated plasma lipid peroxides at the onset of nonaffective psychosis. *Biological Psychiatry*.

[8] Yao J. K., Reddy R. D., Van Kammen D. P. (1999). Human plasma glutathione peroxidase and symptom severity in schizophrenia. *Biological Psychiatry*.

[9] Kaur T., Cadenhead K. S. (2010). Treatment implications of the schizophrenia prodrome. *Current Topics in Behavioral Neurosciences*.

[10] Noto C., Ota V. K., Gadelha A. (2015). Oxidative stress in drug naïve first episode psychosis and antioxidant effects of risperidone. *Journal of Psychiatric Research*.

[11] Sarandol A., Sarandol E., Acikgoz H. E., Eker S. S., Akkaya C., Dirican M. (2015). First-episode psychosis is associated with oxidative stress: effects of short-term antipsychotic treatment. *Psychiatry and Clinical Neurosciences*.

[12] Salzano S., Checconi P., Hanschmann E.-M. (2014). Linkage of inflammation and oxidative stress via release of glutathionylated peroxiredoxin-2, which acts as a danger signal. *Proceedings of the National Academy of Sciences of the United States of America*.

[13] Sánchez A., Calpena A. C., Clares B. (2015). Evaluating the oxidative stress in inflammation: role of melatonin. *International Journal of Molecular Sciences*.

[14] Haring L., Koido K., Vasar V. (2015). Antipsychotic treatment reduces psychotic symptoms and markers of low-grade inflammation in first episode psychosis patients, but increases their body mass index. *Schizophrenia Research*.

[15] WHO (1990). *ICD—International List of Causes of Death, ICD-10*.

[16] Kay S. R., Fiszbein A., Opler L. A. (1987). The positive and negative syndrome scale (PANSS) for schizophrenia. *Schizophrenia Bulletin*.

[17] Kaldur T., Kals J., Ööpik V. (2014). Effects of heat acclimation on changes in oxidative stress and inflammation caused by endurance capacity test in the heat. *Oxidative Medicine and Cellular Longevity*.

[18] Raukas M., Rebane R., Mahlapuu R. (2012). Mitochondrial oxidative stress index, activity of redox-sensitive aconitase and effects of endogenous anti- and pro-oxidants on its activity in control, Alzheimer's disease and Swedish Familial Alzheimer's disease brain. *Free Radical Research*.

[19] Kapczinski F., Frey B. N., Andreazza A. C., Kauer-Sant'Anna M., Cunha Â. B. M., Post R. M. (2008). Increased oxidative stress as a mechanism for decreased BDNF levels in acute manic episodes. *Revista Brasileira de Psiquiatria*.

[20] Hardingham G. E., Do K. Q. (2016). Linking early-life NMDAR hypofunction and oxidative stress in schizophrenia pathogenesis. *Nature Reviews Neuroscience*.

[21] Martínez-Cengotitabengoa M., Mac-Dowell K. S., Leza J. C. (2012). Cognitive impairment is related to oxidative stress and chemokine levels in first psychotic episodes. *Schizophrenia Research*.

[22] Bitanihirwe B. K. Y., Woo T.-U. W. (2011). Oxidative stress in schizophrenia: an integrated approach. *Neuroscience and Biobehavioral Reviews*.

[23] Flatow J., Buckley P., Miller B. J. (2013). Meta-analysis of oxidative stress in schizophrenia. *Biological Psychiatry*.

[24] Leza J. C., García-Bueno B., Bioque M. (2015). Inflammation in schizophrenia: a question of balance. *Neuroscience and Biobehavioral Reviews*.

[25] Nagano T., Mizuno M., Morita K., Nawa H. (2015). Pathological implications of oxidative stress in patients and animal models with schizophrenia: the role of epidermal growth factor receptor signaling. *Current Topics in Behavioral Neurosciences*.

[26] Sarafian T. A., Magallanes J. A. M., Shau H., Tashkin D., Roth M. D. (1999). Oxidative stress produced by marijuana smoke. *American Journal of Respiratory Cell and Molecular Biology*.

[27] Chiu C.-T., Wang Z., Hunsberger J. G., Chuang D.-M. (2013). Therapeutic potential of mood stabilizers lithium and valproic acid: beyond bipolar disorder. *Pharmacological Reviews*.

[28] Lee S.-Y., Lee S.-J., Han C., Patkar A. A., Masand P. S., Pae C.-U. (2013). Oxidative/nitrosative stress and antidepressants: targets for novel antidepressants. *Progress in Neuro-Psychopharmacology and Biological Psychiatry*.

