# Supplementary material for: Antipsychotic Treatment Reduces Indices of Oxidative Stress in First-Episode Psychosis Patients
Source: Oxid Med Cell Longev. 2016 Jul 27;2016:9616593. doi: 10.1155/2016/9616593 (PMC4978850; doi:10.1155/2016/9616593)
Supplement: Supplementary file 1 — The following oxidative stress markers and other measured variables were included in Spearman's rank correlation analysis: serum total antioxidant capacity (TAC), total level of peroxides (TPX), oxidative stress index (OSI), methionine (Met), oxidized methionine (Met-SO), ratio oxidized methionine to methionine (Met-SO/Met), pro- and anti-inflammatory cytokines of the interleukin family (IL-1α, IL-1β, IL-2, IL-4, IL-6, IL-8, IL-10), interferon gamma (IFNγ), vascular endothelial growth factor (VEGF), endothelial growth factor (EGF), tumour necrosis factor-alpha (TNF-α), body mass index (BMI), and Positive and Negative Syndrome Scale (PANSS) total score. Supplementary tables 1-2 demonstrate only significant correlation coefficients of the above-mentioned parameters with OxS markers (TAC, TPX, OSI) in antipsychotic-naïve FEP patients' group. Supplementary tables 3-5 present only significant negative or positive correlation coefficients of the above-mentioned parameters with OxS markers (TAC, TPX, OSI) in FEP patients' group after 7-months treatment with antipsychotics. [file 9616593.f1.pdf]

Supplementary Material

Antipsychotic treatment reduces indices of oxidative stress in first episode psychosis patients

Kärt Kriisa (MSc), Liina Haring (MD), Eero Vasar (PhD), Kati Koido (PhD), Sven Janno (PhD), Veiko Vasar (PhD), Kersti Zilmer (PhD), Mihkel Zilmer (PhD)

*Corresponding Author: Kärt Kriisa, E-mail: kkriisa@gmail.com

The following oxidative stress markers and other measured variables were included in Spearman's rank correlation analysis: serum total antioxidant capacity (TAC), total level of peroxides (TPX), oxidative stress index (OSI), methionine (Met), oxidized methionine (Met-SO), ratio oxidized methionine to methionine (Met-SO/Met), pro- and anti-inflammatory cytokines of the interleukin family (IL-1α, IL-1β, IL-2, IL-4, IL-6, IL-8, IL-10), interferon gamma (IFNγ), vascular endothelial growth factor (VEGF), endothelial growth factor (EGF), tumour necrosis factor-alpha (TNF-α), body mass index (BMI), and Positive and Negative Syndrome Scale (PANSS) total score. Supplementary tables 1-2 demonstrate only significant correlation coefficients of the above-mentioned parameters with OxS markers (TAC, TPX, OSI) in antipsychotic-naïve FEP patients' group. Supplementary tables 3-5 present only significant negative or positive correlation coefficients of the above-mentioned parameters with OxS markers (TAC, TPX, OSI) in FEP patients' group after 7-months treatment with antipsychotics.

Supplementary Table 1: Significant correlation coefficients between total level of peroxides (TPX) and measured parameters in antipsychotic-naïve first-episode psychosis (FEP) patients.

Parameters	Spearman ρ	*p*-value
BMI	0,26	0,03
IL-1β	0,26	0,03
EGF	0,30	<0,01
OSI	0,90	<0,0000001

BMI: body mass index; IL-1β: interleukin 1 beta; EGF: endothelial growth factor; OSI: oxidative stress index.

Supplementary Table 2: Significant correlation coefficients between serum total antioxidant capacity (TAC) and measured parameters in antipsychotic-naïve first-episode psychosis (FEP) patients.

Parameters	Spearman ρ	*p*-value
TNFα	0,26	0,03
IL-1α	0,24	0,04

IL-1β	0,25	0,04

TNF-α: tumour necrosis factor alpha; IL-1α: interleukin 1 alpha; IL-1β: interleukin 1 beta.

Supplementary Table 3: Significant correlation coefficients between oxidative stress index (OSI) and measured parameters in antipsychotic-naïve first-episode psychosis (FEP) patients.

Parameters	Spearman ρ	*p*-value
BMI	0,23	0,049
EGF	0,24	0,04
TPX	0,90	<0,0000001

BMI: body mass index; EGF: endothelial growth factor; TPX: total level of peroxides.

Supplementary Table 4: Significant correlation coefficients between total antioxidant capacity (TAC) and measured parameters after 7-months antipsychotic treatment, in first-episode psychosis (FEP) patients group.

Parameters	Spearman ρ	*p*-value
BMI	0,31	<0,01
OSI	-0,31	<0,01

BMI: body mass index; OSI: oxidative stress index.

Supplementary Table 5: Significant correlation coefficients between oxidative stress index (OSI) and measured parameters after 7-months antipsychotic treatment.

Parameters	Spearman ρ	*p*-value
Treatment	-0,28	0,02
PANSS3	0,24	0,04
IL-6	0,25	0,04
TAC	-0,31	<0,01

Treatment: 7 months of treatment with antipsychotics; PANSS3: general score of psychopathology; IL-6: interleukin 6; TAC: total level of peroxides.
